# Development of a Unified IoT Platform for Assessing Meteorological and Air Quality Data in a Tropical Environment

**DOI:** 10.3390/s24092729

**Published:** 2024-04-25

**Authors:** David Kairuz-Cabrera, Victor Hernandez-Rodriguez, Olivier Schalm, Alain Martinez, Pedro Merino Laso, Daniellys Alejo-Sánchez

**Affiliations:** 1 Faculty of Electrical Engineering, Central University Marta Abreu of Las Villas (UCLV), Santa Clara 54830, Cuba; dkairuz@uclv.cu (D.K.-C.); ecna.baggio@gmail.com (V.H.-R.); amguardia@uclv.edu.cu (A.M.); daniellysas@uclv.edu.cu (D.A.-S.); 2 Antwerp Maritime Academy (AMA), 2030 Antwerp, Belgium; 3 French Maritime Academy (ENSM), 76600 Le Havre, France; pedro.merino-laso@supmaritime.fr; 4 Arts et Métiers Institute of Technology, École navale, IRENAV EA 3634, BCRM de Brest, CC 600, 29240 Brest cedex 9, France

**Keywords:** air quality, internet of things, monitoring system, Node-RED, Grafana, TTGO TBeam, weather station, low-cost sensors

## Abstract

In developing nations, outdated technologies and sulfur-rich heavy fossil fuel usage are major contributors to air pollution, affecting urban air quality and public health. In addition, the limited resources hinder the adoption of advanced monitoring systems crucial for informed public health policies. This study addresses this challenge by introducing an affordable internet of things (IoT) monitoring system capable of tracking atmospheric pollutants and meteorological parameters. The IoT platform combines a Bresser 5-in-1 weather station with a previously developed air quality monitoring device equipped with Alphasense gas sensors. Utilizing MQTT, Node-RED, InfluxDB, and Grafana, a Raspberry Pi collects, processes, and visualizes the data it receives from the measuring device by LoRa. To validate system performance, a 15-day field campaign was conducted in Santa Clara, Cuba, using a Libelium Smart Environment Pro as a reference. The system, with a development cost several times lower than Libelium and measuring a greater number of variables, provided reliable data to address air quality issues and support health-related decision making, overcoming resource and budget constraints. The results showed that the IoT architecture has the capacity to process measurements in tropical conditions. The meteorological data provide deeper insights into events of poorer air quality.

## 1. Introduction

Air is recognized as a global threat to human health by the World Health Organization (WHO). It also poses challenges to achieving the Sustainable Development Goals of the United Nations as outlined in the 2030 agenda, in particular SDG 3 [1]. The adverse impact of poor air quality extends to various respiratory conditions, such as asthma and chronic obstructive pulmonary disease (COPD) [2,3]. In addition, it has been linked to the development of diseases such as diabetes, lung cancer, cardiovascular problems, and an increased prevalence of psychological disorders affecting mental health [4,5]. The detrimental effects of air pollution go beyond human health, as it is associated with the occurrence of acid rain and smog. Additionally, it can harm animals and cause damage to vegetation and food crops [6,7].

Air quality monitoring typically relies on costly stationary or mobile stations, needing frequent calibration and skilled operators for precise data collection. Yet, these setups often lack spatial coverage, hindering accurate measurements, notably in economically constrained regions. To tackle this, affordable air quality sensors have emerged, enabling deployment in resource-limited areas [8]. However, it is vital to acknowledge their reduced accuracy compared to professional stations. Consequently, they are mainly used to detect specific patterns in pollution dynamics, such as pinpointing pollution events, rather than tasks demanding precise measurements. This innovation addresses accessibility issues but underscores the importance of considering sensor limitations in air quality assessments [9,10].

Meteorology plays a pivotal role in understanding and managing air quality, as environmental conditions greatly influence the concentrations of pollutants in the atmosphere. Meteorological factors such as temperature, humidity, wind speed, and atmospheric pressure directly impact the dispersion and transformation of pollutants emitted from various sources such as industrial activities, transportation, and natural phenomena such as wildfires [11]. For instance, stagnant atmospheric conditions can lead to the buildup of pollutants in urban areas, exacerbating air quality issues and posing serious health risks to populations. Conversely, strong winds can disperse pollutants over large areas, affecting regions far from their sources [12]. By integrating meteorological data into air quality monitoring and forecasting systems, policymakers and environmental agencies can better assess pollution levels, anticipate potential air quality events, and implement timely interventions to mitigate the adverse impacts on public health and the environment. Therefore, meteorology serves as a critical tool in understanding the complex interactions between atmospheric dynamics and air quality essential for developing effective strategies to protect human health and ecosystems from the detrimental effects of air pollution [13].

Unfortunately, existing weather stations in many low-income countries are affected by the same issues as air quality stations: high cost of equipment and lack of trained personnel for maintenance and operation [13]. A possible solution to this lack is the integration of low-cost automatic weather stations with air quality systems powered by the internet of things (IoT). By combining real-time meteorological data with air quality measurements, these systems provide a comprehensive understanding of atmospheric conditions and pollutant levels [14]. IoT-enabled sensors can continuously collect data on temperature, humidity, wind speed, atmospheric pressure, and various pollutants, allowing for precise monitoring and analysis. This integration facilitates early detection of air quality issues, enabling proactive measures to be taken to protect public health and the environment [15,16].

The scalability and flexibility of IoT technology allow for the deployment of low-cost sensor networks in diverse geographical locations, far from urban areas, providing comprehensive coverage and enhancing the accuracy of environmental assessments [17]. Additionally, the remote monitoring capabilities of these systems enable efficient data collection and analysis, reducing the need for manual intervention and minimizing operational costs [18,19]. Overall, the integration of automatic weather stations with air quality systems through IoT technology represents a powerful approach to environmental monitoring, offering timely insights and enabling informed decision making for sustainable development and resource management [20,21].

The use of low-cost embedded hardware in a tropical climate poses a unique additional challenge for the technology developed to monitor air quality [22]. The high temperatures and humidity levels prevalent in tropical regions can affect the performance and reliability of monitoring equipment. These conditions can lead to accelerated degradation of sensors, reducing their accuracy and lifespan. Additionally, intense rainfall and thunderstorms common in tropical climates may disrupt communication systems and power supply to monitoring stations, hindering data transmission and collection [23,24]. To overcome these challenges, researchers and engineers need to design new sensor systems that can withstand the rigors of tropical climates [23,24,25]. By developing tailored solutions that account for the specific challenges of tropical climates, researchers and policymakers can ensure the effectiveness and reliability of air quality monitoring systems in these regions, ultimately supporting efforts to safeguard human health and the environment.

Cuban government agencies grapple with limited resources in monitoring air quality, lacking continuous automated devices. They rely on localized systems without remote capabilities, resulting in irregular manual sample collection and analysis. This situation limits long-term data accessibility, hindering comprehensive studies on historical trends. Meanwhile, at the Central University “Marta Abreu” of Las Villas (UCLV), collaboration with international partners has led to the development of systems for measuring air quality variables [25,26] and meteorological parameters [27]. These systems are based on open technologies and low-cost calibrated sensors, designed to provide valuable information for decision making [28,29]. However, integration and interoperability issues persist, causing fragmented data collection and analysis.

To address the abovementioned problems, the present research aims to create a unified IoT platform gathering data from diverse sources for understanding tropical environmental conditions. This paper presents the designs of a versatile monitoring system utilizing IoT technology to analyze air quality and climate parameters suitable for urban or remote areas. It introduces an affordable IoT solution for measuring air quality and weather conditions, particularly focusing on their interrelation in tropical environments.

## 2. Background

Several companies have recognized the need for integrated solutions in monitoring air quality and meteorological parameters, simplifying IoT technology for wider adoption [30]. Well-known systems such as AQMesh [31] and Libelium’s Smart Environment [32] or Smart Environment Pro [33], utilizing cost-effective sensors, have undergone extensive real-world testing and deployment. Some of these systems are modular, allowing users to choose specific sensors to address their particular issues.

Alternatively, users can combine open-source building blocks and build their own system. Academic research delves deeper into this area, utilizing various platforms and technologies, as presented in Table 1. These studies contribute to a more profound understanding, forming the basis for further investigations. This approach requires greater technical background knowledge on the part of the user. It is clear from Table 1 that temperature and humidity are the most commonly monitored parameters in IoT technologies [21]. Wind direction and speed, solar radiation, atmospheric pressure, and precipitation are also included when meteorological parameters are to be measured. Parameters such as VOC (Volatile Organic Compounds), PM (Particulate Matter), O_3_ (Ozone), SO_2_ (Sulfur dioxide), CO_2_ (Carbon dioxide), CO (Carbon monoxide), and NO_2_ (Nitrogen dioxide) are the base of most air quality monitoring systems. For this kind of research, ESP32, NodeMCU, and Raspberry Pi are widely used as processing units, either independently or in conjunction with add-on boards that extend their communication capabilities [34]. Many systems that can be found in the literature measure either air quality or weather parameters. Only a few have made the integration of both, and in some cases, wind speed and direction are not taken into account. The few publications that integrate all these parameters reflect the importance of the joint measures for posterior analysis.

### 2.1. Communication Technologies

The IoT relies on efficient communication technology for seamless device connectivity and data exchange. WiFi, GSM, and LoRa are commonly used (see Table 1), each with distinct advantages [49]. WiFi offers high-speed data transfer suitable for real-time applications such as home automation [50]. However, WiFi is limited to short distances. GSM provides wide coverage, ideal for remote areas and long-range applications such as asset tracking [50], but its usage depends on the availability of telecommunication providers for long-range functionality. LoRa specializes in long-range, low-power communication, suitable for widespread deployment in sectors such as agriculture, smart metering, and logistics. Its ability to pass through obstacles and send signals over several kilometers, coupled with its low-cost infrastructure, makes LoRa an attractive option for IoT deployments that require long-range connectivity and extended battery life [51]. Therefore, the LoRa protocol emerges as a viable alternative capable of facilitating communication over longer distances and in areas where GSM coverage is not available. For this reason, LoRa has been selected as the optimal communication technology for this work.

### 2.2. Software Platforms for IoT

Software IoT platforms have transformed connectivity, managing vast data volumes from billions of devices, crucial for informed decision making. These platforms equip the infrastructure to acquire, process, store, and analyze IoT data. Common tools for monitoring air quality and weather include ThingSpeak, The Things Network, Node-RED, and Grafana [52]. ThingSpeak offers an open-source platform for defining data channels and capturing data from various sources using HTTP or MQTT protocols, while The Things Network utilizes LoRa technology for decentralized data acquisition [53]. Although ThingSpeak and The Things Network offer significant capabilities, they are not accessible without cost. In contrast, Node-RED excels in data acquisition and processing, InfluxDB in storage efficiency, and Grafana in data visualization. Combining these tools creates a flexible IoT software platform free of cost and able to meet specific research needs.

Besides the combination of Node-RED, InfluxDB, and Grafana, other combinations of freely available software blocks are also available to build an IoT platform. In addition to InfluxDB, Prometheus and QuestDB can also be used to process time series data. Prometheus is widely used for monitoring, while QuestDB shows reliable performance despite its newness. InfluxDB, though with weaker performance, remains a viable option because it is easy to program and has adequate security capabilities [54,55]. Node-RED has limited possibilities to visualize information. Instead, Grafana, Kibana, or Metabase can be integrated in the IoT software platform. However, Kibana and Metabase have limitations compared to Grafana’s flexibility, data handling, and customization options, making Grafana preferable in scalable scenarios [56,57,58].

## 3. Unified IoT Platform System Design

The preceding section provided an overview of the possibilities to construct a low-cost IoT platform, detailing some essential decisions related to the use and selection of specific building blocks. The following section gives a detailed description of the constructed platform.

### 3.1. Hardware

**Figure 1:**
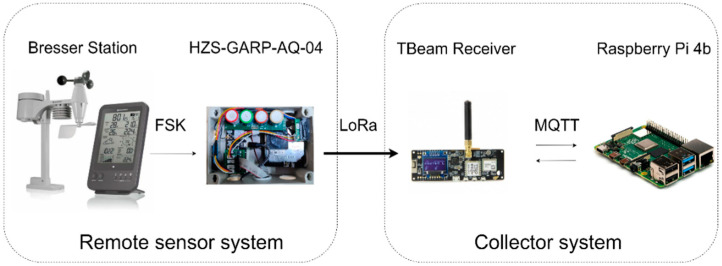
Overview of the hardware architecture of the IoT monitoring system.

Figure 1 shows the proposed hardware architecture. It consists of a “remote sensor system” and a “collector system”. The remote sensor system consists of a Bresser 5-in-1 weather station that measures the meteorological parameters and an in-house developed sensor box (i.e., HZS-GARP-AQ-04) containing an ESP32-based development board named TTGO TBeam that collects all acquired data and sends them to the collector system using LoRa communication. The collector system contains the same ESP32 (i.e., TBeam Receiver) to receive all data, which is connected to a Raspberry Pi used for edge computing.

#### 3.1.1. Remote Sensor System

The Bresser weather station model 7,002,510 consists of a device containing the external sensors that measure five different meteorological parameters (Table 2) and a base station [59]. Both devices are powered by three 1.5 V AA batteries that allow continuous operation for several months. The external sensor is programmed to capture data at 45-s intervals and transmits this information wirelessly to the base station using frequency shift keying (FSK). Upon transmission, the TBeam board within the HZS-GARP-AQ-04 system intercepts these measurements for further processing.

The system used for air quality measurements is a variation of a previously developed sensor box (i.e., HZS-GARP-AQ-04) [25]. It utilizes the TTGO TBeam development board as its foundation. That board is equipped with an ESP32 microcontroller, PSRAM for storage, and WiFi and Bluetooth capabilities for short-distance communication. Additionally, a SX1276 Radio module, enabling LoRa or FSK communication, facilitates long-range communication. The sensors deployed in the HZS-GARP-AQ-04 (Table 3) were calibrated using low-cost methods as described elsewhere [28,29]. The specifications for all the sensors’ environmental operating conditions suggest that they can be used in a tropical environment.

**Figure 2:**
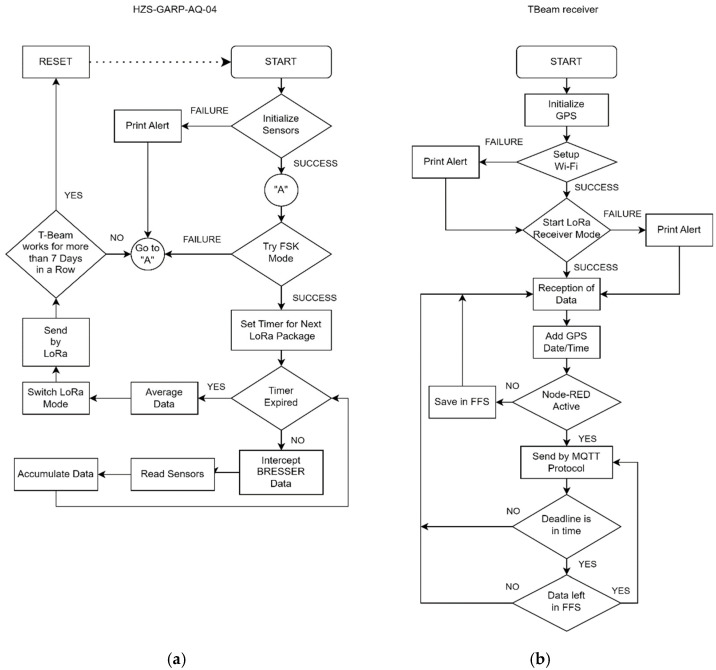
Flowchart used to collect, process, send, and receive measurements for (**a**) the HZS-GARP-AQ-04 and (**b**) the TBeam Receiver.

The TBeam inside the HZS-GARP-AQ-04 receives, through wireless FSK communication, the data sent by the Bresser station. The decoding process for extracting information transmitted by the Bresser station and acquiring it with a TBeam board is detailed in [27]. The duty cycle of the TBeam board in the HZS-GARP-AQ-04 handles the measurements of the meteorological station and the sensors interconnected directly to it which send data at different rates. In addition, it calculates the average values for each sampling period (Figure 2a). The flowcharts in Figure 2a also include a control for communication interruptions and can trigger a board reset if needed.

#### 3.1.2. Collector System

The TTGO TBeam-based receiver collects the information from the “remote sensor system” through LoRa protocol using an 868 MHz frequency. MQTT (Message Queuing Telemetry Transport) protocol is used to establish a connection between the TBeam Receiver and the Raspberry Pi using the Mosquitto broker. MQTT is a lightweight messaging protocol designed for efficient communication between IoT devices and resource-constrained environments. The TTGO TBeam board acts as a sensor or actuator device, while the Raspberry Pi acts as a central host. The Mosquitto broker acts as an intermediary for message exchange.

**Figure 3:**
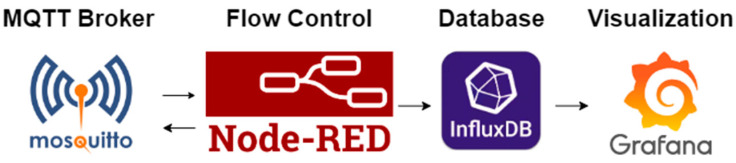
Software architecture in the Raspberry Pi used to collect and visualize data.

The Raspberry Pi 4 Model B, receiving data via MQTT from the TBeam Receiver, boasts a Quad-core 64-bit ARM Cortex (Cambridge, UK) processor clocked at 1.5 GHz. It packs 2 GB of RAM and operates on a 32 GB SD card. It is connected to the TBeam Receiver’s network for data exchange by Ethernet and WiFi. Its robust computational power is vital for future edge computing and prediction algorithms, addressing challenges inherent in IoT device computing, which demand adherence to strict requirements [60,61]. The Raspberry Pi implements an open-source IoT architecture comprising back-end components (Node-RED, MQTT Broker, and InfluxDB) and a front-end component (Grafana), as illustrated in Figure 3. Node-RED acquires and processes received information, storing it in InfluxDB, and presenting it on a Grafana dashboard.

In the Raspberry Pi, the logic followed after the capture of each data packet consists of adding a temporary identifier to the frame before sending it to an “eclipse-mosquitto” broker via MQTT. In case of an error during this process, the frame is saved in the internal storage (SPIFFS), to be returned to the received state by LoRa. If the sending is successful, it is finally checked for unsent iterations before returning to the receiving state of the system. Figure 2b shows the flow followed by this device. The TBeam Receiver acts as a gateway, is located indoors, and must be connected to the same network as the Raspberry Pi to achieve data transfer. To enhance its protection and portability, a case was fabricated using an Ender 5 3D printer.

**Figure 4:**
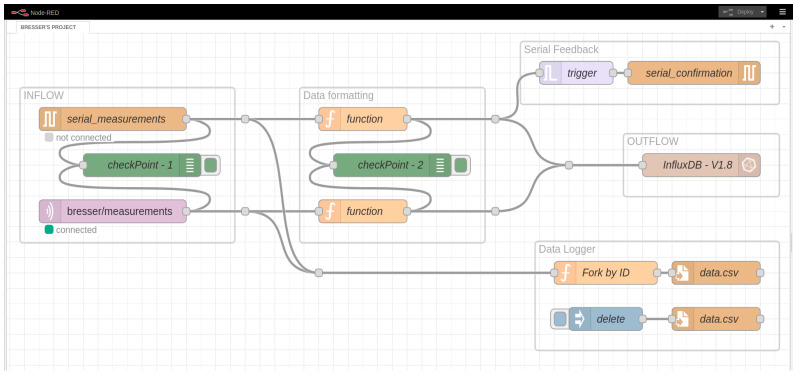
Node-RED flow for data acquisition and process.

The IoT platform Node-RED utilizes the MQTT broker to process data from the remote sensor system. This visual tool employs flow programming, easing connectivity among hardware devices, APIs, and online services [62,63]. Data variables are parsed and organized into an ordered pattern within the InfluxDB database. Figure 4 illustrates the control flow ensuring data packet integrity, segmented into functional blocks for clarity.

The flow features two input types: “serial” for direct USB connection and “MQTT” utilizing the broker as an intermediary. Data formatting and validation occur in the “data formatting” block, producing JSON output structured with a UNIX timestamp compatible with InfluxDB. Serial input confirms data reception via a feedback node, while MQTT relies on broker mechanisms and QoS for integrity.

Timestamp-based identification maintains data integrity for transmission resumption. Incoming data is stored in a .CSV file for easy access and inspection facilitated by the “data logger” block. Real-time monitoring allows anomaly detection within the system architecture, aiding in data stream filtering for further analysis in external programs like MATLAB, enhancing data processing capabilities.

Grafana takes information from a database in InfluxDB. The dashboard was developed to offer a user-friendly interface, presenting key information and aiding in data comprehension. It comprises two primary sections: meteorological variables and air quality parameters. Each section displays the latest measurements. Furthermore, the Grafana dashboard includes historical graphs that enable users to track the trends and behaviors of variables over time. The data visualization gives insight to the user about the environmental dynamics, assisting users in identifying data patterns, anomalies, or trends.

## 4. Experimental

To assess the performance of the unified IoT platform system design outlined in the previous section, a 15-day experiment was conducted in a real outdoor setting. For outdoor testing, the remote sensor system was installed on the roof of the Faculty of Electrical Engineering at UCLV, Santa Clara, Cuba. Positioned atop a four-story building, the surroundings were free from obstructions, such as taller buildings or trees, that might impede airflow. The Collector System was located inside an office on the first floor of that building.

The Smart Environment Pro is an IoT solution manufactured by Libelium and was used as a reference for the IoT platform. In this case, the Libelium device contained the Alphasense sensors for CO, O_3_, NO_2_, and SO_2_ and were connected on sockets A, B, C, and F, respectively. In this model, the sensor in socket D was always reserved for PM_1_, PM_2.5_, and PM_10_ using an OPC-N3 optical particle counter. The temperature, humidity, and pressure probe was on socket E with a BME280 sensor. It had the ATmega 1281 microcontroller as core, working at a frequency of 14.7456 MHz, and allowed an SD card module up to 2 GB. The device incorporated a Real Time Clock (RTC) that allowed the Waspmote sensor platform to go to Deep Sleep and Hibernate modes for energy saving. It contained a rechargeable lithium-ion battery of 3.7 V (nominal voltage) and a capacity of 6600 mAh. It included a rigid solar panel of 6 W. This device can be programmed similarly to an Arduino using the Waspmote IDE, developed and maintained by the company [64].

**Figure 5:**
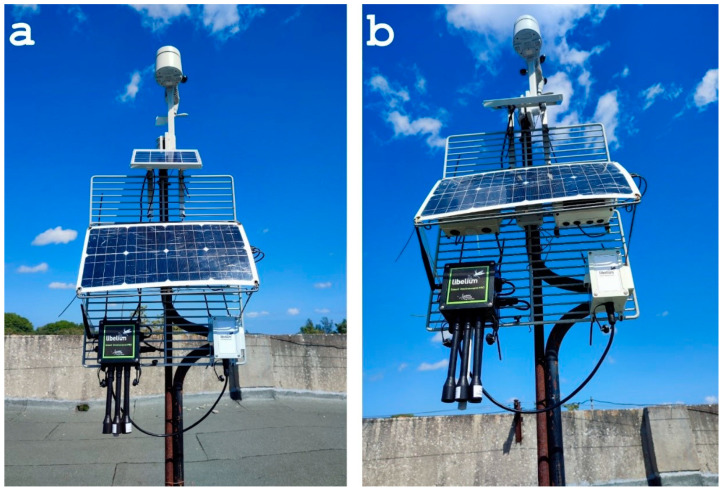
The sensor system and the reference system by Libelium deployed on the roof of the building of the Faculty of Electrical Engineering, Santa Clara, Cuba. (**a**) Front view showing the Libelium system attached at the bottom of the grid. (**b**) The setup seen in frog perspective showing the IoT sensor system underneath the large solar panel.

Figure 5 depicts the configuration for the comparative study, showcasing how two distinct air quality measurement systems monitor the same environmental conditions simultaneously. The Smart Environment Pro instrument of Libelium, accompanied by a smaller solar panel, was positioned on a mast at a height of 1.50 m from the roof. For the remote sensor system, the HZS-GARP-AQ-04 was positioned 30 cm above the reference instrument, at a height of approximately 1.80 m. That system was connected to the larger solar panel shown in Figure 5. The Bresser station was installed on top of the mast at a height of 2.10 m, facing north. The placement of both systems on the rooftop ensured exposure to the environmental conditions of interest, facilitating the evaluation of their performance and reliability.

Data cleaning and data visualization for analyses were performed with the software MATLAB. To evaluate and compare signals obtained from two different measuring systems, several parameters are commonly used, such as the coefficient of determination (R^2^), cross-correlation, root mean square error (RMSE), mean square error (MSE), and mean absolute error (MAE). In particular, in air quality studies, it has been observed that the most commonly used parameters for comparison with reference methods are the RMSE, the coefficient of determination (R^2^), the cross-correlation, and the comparison of the measuring range (Mean + stdev) of both methods [13,65,66,67,68]. 

## 5. Results

**Figure 6:**
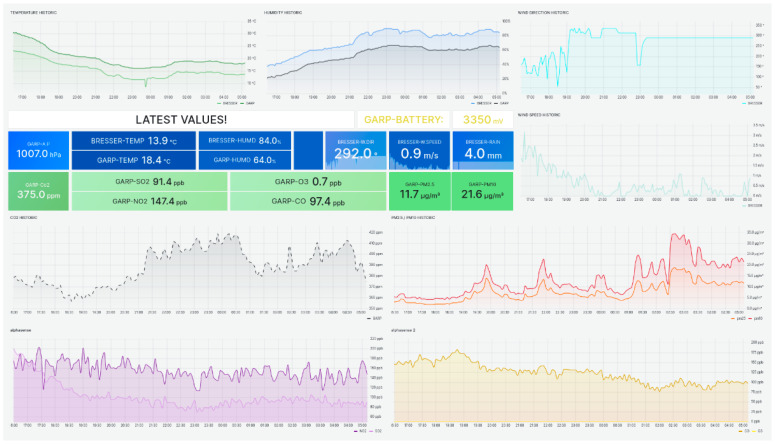
Grafana dashboard for the visualization of output variables of the unified IoT platform.

The Grafana dashboard, shown in Figure 6, indicates that it is possible to collect all data and visualize their dynamic patterns in graphs in real time. This facilitates the analysis of the parameter’s dynamics and allows for faster action when unforeseen events occur. By integrating weather and air quality data sources, the dashboard provides a global view of the rooftop environment. The Grafana dashboard appears to be a valuable tool for visualizing and interpreting the local air quality, allowing for informed decisions.

**Figure 7:**
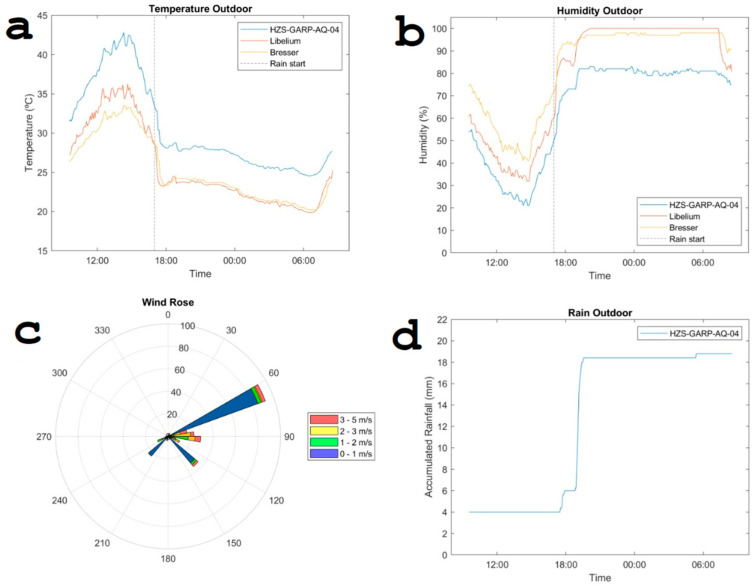
Temperature and relative humidity collected by the remote sensor system and the reference system by Libelium, complemented by meteorological information from the Bresser station.

A comparative analysis was conducted between the remote sensor system and the reference instrument for temperature and relative humidity. Figure 7 illustrates a “cold start” scenario where both systems were suddenly switched on. It shows the details of a larger measurement campaign where the black dashed line indicates the start of a rain event. Throughout the 15-day observation period, a cumulative rainfall of 18.8 mm was recorded (see Figure 7d). Wind speeds remained generally subdued, peaking at 5 m/s, with occasional calm periods. The predominant wind direction was primarily east. This information is shown in the wind rose in Figure 7c. The pattern of the temperature and relative humidity dynamics obtained from the different sensors is similar except for a vertical shift. This means that the calibration of the sensors was not perfect.

**Figure 8:**
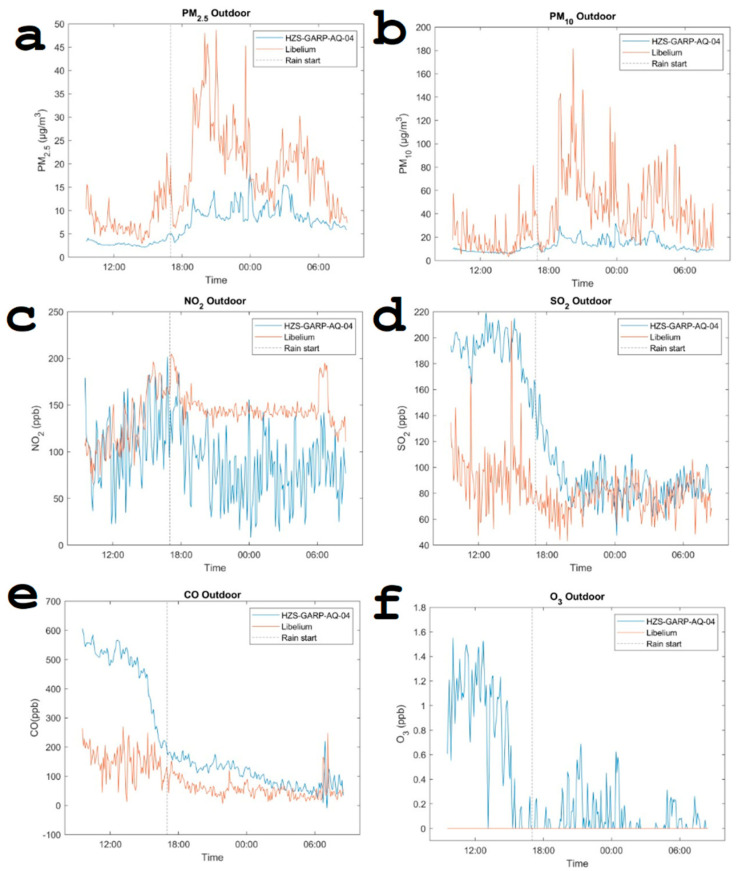
Dynamic patterns of pollutants observed on 5 and 6 March 2024, just before and after rainfall, following the activation of the unified IoT platform and the Libelium system at the same location.

Another comparative analysis was conducted between the remote sensor system and the reference instrument for the pollutants PM_2.5_, PM_10_, NO_2_, SO_2_, CO, and O_3_. Figure 8 illustrates the impact of a “cold start” on the accuracy of measurements obtained with the Alphasense electrochemical sensors. The deliberate inclusion of a transition period was intended to evaluate the performance of the Alphasense sensors under varying conditions, providing insights into their response and stability over time [65]. The analysis reveals complex behavior in the measured parameters, characterized by pronounced peaks on top of a slowly fluctuating background. For most peaks, there is synchrony between both systems. However, the calibration of the sensors is, for both systems, fundamentally different. For particulate matter, there is an observed increase in concentration when it is raining, possibly because the sensor misidentifies small water droplets as solid particles. A sudden drop in sulfur dioxide (SO_2_) is observed when it starts to rain, likely due to its dissolution in water. Notably, the Libelium system failed to record any data for ozone levels, and similarly, the remote sensor system detected ozone concentrations that were barely above the sensor’s threshold. Incorporating meteorological information offers a significant benefit by providing additional context, which is crucial for interpreting sudden shifts in dynamic patterns. This extra layer of data helps elucidate the environmental factors influencing these changes, enhancing the analysis and understanding of the data collected.

To delve deeper into how the measurements from both systems align, various comparative analyses were conducted. The outcomes of these studies are consolidated in Table 4. The highest congruence between the HZS-GARP-AQ-04 and the Libelium system measurements, as illustrated through linear regression, was seen in the temperature and humidity data, both achieving an R^2^ value of 0.98. This high level of correlation was anticipated because both systems utilize BME280 sensors for measuring these parameters. Nonetheless, despite this high correlation, a notable difference in the absolute quantities measured by each system was observed.

**Figure 9:**
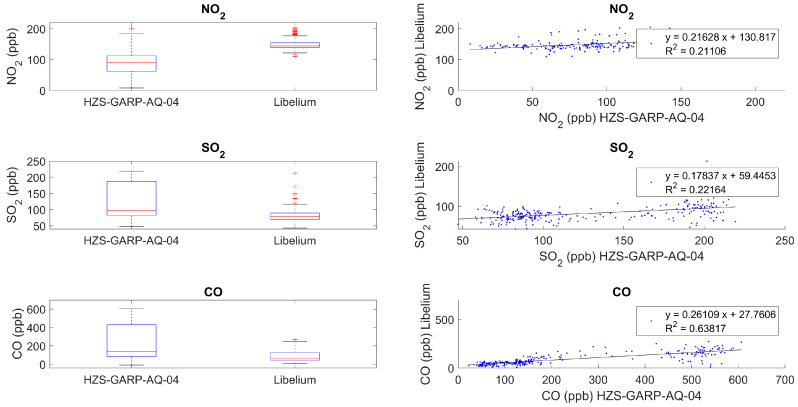
Relation between the measurements of HZS-GARP-AQ-04 and Libelium Smart Environment Pro for gaseous pollutants.

**Figure 10:**
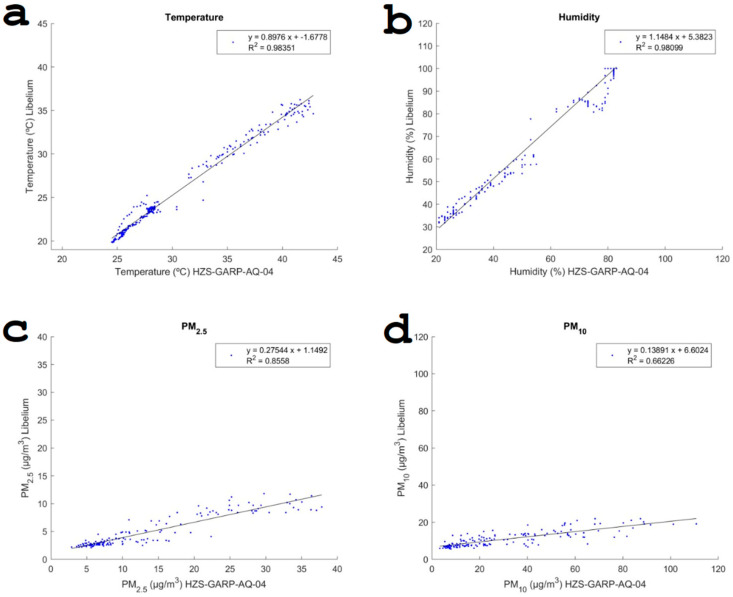
Relation between the measurements of HZS-GARP-AQ-04 and Libelium Smart Environment Pro for Temperature, RH, and particulate matter.

Despite the usage of the same Alphasense gas sensors and their calibration by the manufacturer, SO_2_, NO_2_, and CO measurements showed no clear correlation between both systems. The best, but not good, fit regarded CO (R^2^ = 0.63) measurements, while SO_2_ (R^2^ = 0.22) and NO_2_ (R^2^ = 0.21) displayed poor performance when compared with Libelium (Figure 9). O_3_ showed relatively low values for the HZS-GARP-AQ.04 with an average of 0.3 ppb and a maximum of 1.55 ppb—concentrations that fell below the detection limit of Libelium (Figure 10).

For particulate matter, PM_2.5_ showed a high fit (R^2^ = 0.85) between the Nova SDS011 sensor (Nova Fitness, Jinan, China) used for HZS-GARP-AQ-04 and the Alphasense OPC-N3 in the Libelium system. A relatively poor fit was achieved for the PM_10_ concentrations (R^2^ = 0.66). This low coefficient can be explained by the overestimation of the PM_10_ concentrations by OPC-N3 and its predecessor model, OPC-N2 [69].

## 6. Discussion

While extended periods of operation are necessary to confirm its resilience in tropical environments, initial results show no signs of degradation of the hardware components. Leveraging its LoRa communication capability, the platform ensures seamless data transmission over distances of up to 2 km in open fields, with negligible data packet loss. The collector device received 98.2% of the packages sent by the sensor system, demonstrating a low package loss and the correct functioning of the LoRa protocol. These promising findings underscore the platform’s potential for reliable environmental monitoring applications, particularly in challenging conditions prevalent in tropical regions.

In the conducted tests, the Libelium system exhibited a notable autonomy of 48 h utilizing a 6600 mAh battery. Conversely, the HZS-GARP-AQ-04 system demonstrated a battery endurance of 10 h with a 2600 mAh battery. Considering that the Libelium battery possesses a capacity 2.5 times greater than that of the HZS system, it is reasonable to deduce that, under these circumstances, the system equipped with a 6600 mAh battery should provide 25 h. It is worth mentioning that the Libelium system was not engaged in any wireless communication during the test, while the other system was consistently receiving data via FSK or transmitting data through LoRa.

The purpose of incorporating the Libelium system in this study was to conduct a comparative analysis between the measurements obtained from the IoT platform and a commercially available device designed to measure similar air pollutants. It should be emphasized that some of the sensors used by Libelium are similar to the ones in the IoT platform. While Libelium trusts the calibration provided by Alphasense, we also conducted additional calibrations. Differences in the absolute values between the two systems are mainly caused by imperfect calibration, cross-interference (e.g., rain droplet considered as particulate matter), calibration drift, etc. As a result, it is hard to determine what measurements are the most reliable. Therefore, data from the Libelium system were not used as control measurements to adjust the results of the proposed platform.

External factors such as rain can obscure genuine signals in PM sensor readings, making it challenging to isolate these signals accurately. Therefore, post-processing techniques may not be fully effective in removing these external influences. Additionally, the high-frequency components of the signals, including peaks and valleys, are essential in this type of research and should be preserved. As a result, the analysis was carried out using the unfiltered, raw data from the IoT platform. This approach allows the data to be evaluated in their original state, facilitating an examination of sensor performance. By opting not to use post-processing techniques in the current analysis, we maintain the option to implement them in future projects, after gaining a deeper understanding of the platform’s behavior.

In terms of component costs alone, the estimated value of the system is around USD 650, without taking into account factors such as intellectual labor, shipping, and packaging costs. In comparison, existing commercial solutions, such as AQ Mesh, are priced at around USD 3500, while the Libelium Smart Environment Pro system used in this study cost approximately USD 5000.

## 7. Conclusions

The unified IoT platform efficiently handled and displayed data for both meteorological and air quality measurements, demonstrating strong performance under the demanding conditions of a tropical environment, notably high humidity levels and solar radiation, without any system failures. This success illustrates the feasibility of deploying a network of monitoring stations to consistently track environmental parameters on a constrained budget. This study shows that it is possible to monitor key risk parameters that determine the impact of air quality to human health in a developing country.

The comparative analysis between the unified IoT platform developed and the Libelium Smart Environment Pro determined both are able to capture the dynamics of the environmental parameters, making them well suited for air quality monitoring and identifying events causing moments of poorer air quality. Statistical analysis highlighted the differences in performance between the Alphasense sensors used in the HZS-GARP-AQ-04 device and the Libelium system. This is evidenced by lower R-squared values. There are significant differences between the measurements of both systems. Since both systems use the same types of sensors, it is not entirely clear which system is more reliable in determining the absolute amount of the measured quantities. This shows the need for sensor calibration and, in particular, regular in situ calibrations to improve the reliability of the sensor measurements.

Further research is required to improve the reliability of air quality measurements. The sensors used in this study to measure NO_2_, O_3_, SO_2_, and CO are provided by Alphasense, and the manufacturer indicates these sensors generally have a lifespan of about two years. A future study should therefore aim to assess how these sensors perform in a tropical environment, which typically causes a faster degradation of materials and electronics. While the IoT platform generally performed adequately, it is important to implement in situ calibration to maintain high data quality throughout the monitoring campaign. Future deployments will include these calibrations to support accurate decision making and health assessments.

## Figures and Tables

**Table 1:** IoT air quality and meteorological monitoring-related research.

Issue	Air Quality Variables	Meteorological Variables	Microcontrollers and Single-Board Computers	Network	IoT Platform
[35]	No	T, RH, AP, rainfall	Node MCU	WiFi	Web page
[36]	No	T, RH, AP, rainfall, solar intensity	ESP32	WiFi	ThingSpeak
[37]	No	T, RH, rainfall, solar intensity, vibration	Raspberry Pi 3	WiFi	ThingSpeak
[38]	CO, CO_2,_ PM_2.5_	No	Arduino UNO	WiFi	Web page
[39]	O_3,_ NO_2,_ SO_2_, CO	T, RH	Arduino UNO	WiFi	Web page
[40]	NO_2_, CO	T, RH	Node MCU	WiFi	Google Sheet
[16]	CO, PM_1_, PM_2.5_, PM_10_	T, RH	Raspberry Pi 4	WiFi	No
[41]	O_3_	T, RH, AP, rainfall, WS, WD	Arduino Mega	NB-IoT	Grafana
[42]	CO, CO_2_, O_3_, CH_4_, Ammonia, Hydrogen	T, RH, AP, rainfall, WS, WD, UVI	ESP32	GSM	Web page
[43]	CO_2_, NO_2_	T, RH, AP	ESP32	WiFi	Node-RED
[13]	CO_2_, PM_1_, PM_2.5_, PM_10_	T, RH, AP	STM32 ARM Cortex M3 and Node MCU	WiFi	ThingSpeak
[44]	O_3_, NO_2_, SO_2_, CO	T, RH, AP, rainfall, WS, WD	PIC and Raspberry Pi 3	WiFi/GSM	Web page
[45]	O_3_, NOx, PM_2.5_	T, RH, WS, WD	ESP32	LoRa	The Things Network
[46]	PM_2.5_, PM_10_	T, RH	Mediatek LinkIt One	GSM	Web page
[47]	CO, VOC, PM_2.5_, PM_10_	T, RH, AP	Arduino UNO and Node MCU	WiFi	ThingSpeak
[48]	CO, NO_2_, VOC	T, RH, AP	ESP32 LoRa SX1278	LoRa	The Things Network

T: temperature, RH: relative humidity, AP: atmospheric pressure, WS: wind speed, WD: wind direction, UVI: UV index, CH_4_: methane, VOC: volatile organic compound.

**Table 2:** Meteorological variables measured by the Bresser 5-in-1 and the corresponding detection ranges.

Variable	Detection Range
Temperature	−40–80 °C
Relative humidity	0–100%
Rainfall	0–9999 mm
Wind speed	0–180 km/h
Wind direction	0–360°

**Table 3:** Sensors and variables measured by the in-house developed sensor box HZS-GARP-AQ-04.

Sensor	Variable	Interface	Detection Range
Sensirion SCD30	CO_2_	I2C	400–10,000 ppm
Nova SDS011	PM_2.5_/PM_10_	UART	0–999.9 μg/m^3^
BME280	T/RH/AP	I2C	0–100%/−40–85 °C/300–1100 hPa
OX-A431	O_3_	Analog	0–20 ppm
SO2-A4	SO_2_	Analog	0–50 ppm
NO2-A43F	NO_2_	Analog	0–20 ppm
CO-AX	CO	Analog	0–2000 ppm

**Table 4:** Statistical overview of some of the collected parameters with the two monitoring systems supplemented with statistical tests to assess the correlation between both systems.

Variable	HZS-GARP-AQ-04 Mean ± Stdev	Libelium Mean ± Stdev	Cross Correlation	RMSE	R^2^
Temperature (°C)	30 ± 6	26 ± 5	0.99	4.87	0.98
Humidity (%)	65 ± 22	80 ± 26	0.99	15.72	0.98
SO_2_ (ppb)	125 ± 52	82 ± 20	0.47	63.32	0.22
NO_2_ (ppb)	89 ± 38	150 ± 18	0.46	69.74	0.21
CO (ppb)	220 ± 180	86 ± 60	0.77	194.56	0.63
PM_2.5_ (μg/m^3^)	5 ± 3	14 ± 10	0.91	11.40	0.85
PM_10_ (μg/m^3^)	11 ± 4	32 ± 25	0.81	29.88	0.66

## Data Availability

All data used in this study are available upon request.
